# The Role of Calcium–Calcineurin–NFAT Signaling Pathway in Health and Autoimmune Diseases

**DOI:** 10.3389/fimmu.2020.00195

**Published:** 2020-03-10

**Authors:** Yune-Jung Park, Seung-Ah Yoo, Mingyo Kim, Wan-Uk Kim

**Affiliations:** ^1^POSTEC-CATHOLIC Biomedical Engineering Institute, The Catholic University of Korea, Seoul, South Korea; ^2^Division of Rheumatology, Department of Internal Medicine, St. Vincent's Hospital, The Catholic University of Korea, Suwon, South Korea; ^3^Department of Biomedicine & Health Science, College of Medicine, The Catholic University of Korea, Seoul, South Korea; ^4^Division of Rheumatology, Department of Internal Medicine, Gyeonsang National University Hospital, Jinju, South Korea; ^5^Division of Rheumatology, Department of Internal Medicine, The Catholic University of Korea, Seoul, South Korea

**Keywords:** calcium, calcineurin, nuclear factor of an activated T-cell, Ca^2+^ signaling, autoimmune disease

## Abstract

Calcium (Ca^2+^) is an essential signaling molecule that controls a wide range of biological functions. In the immune system, calcium signals play a central role in a variety of cellular functions such as proliferation, differentiation, apoptosis, and numerous gene transcriptions. During an immune response, the engagement of T-cell and B-cell antigen receptors induces a decrease in the intracellular Ca^2+^ store and then activates store-operated Ca^2+^ entry (SOCE) to raise the intracellular Ca^2+^ concentration, which is mediated by the Ca^2+^ release-activated Ca^2+^ (CRAC) channels. Recently, identification of the two critical regulators of the CRAC channel, stromal interaction molecule (STIM) and Orai1, has broadened our understanding of the regulatory mechanisms of Ca^2+^ signaling in lymphocytes. Repetitive or prolonged increase in intracellular Ca^2+^ is required for the calcineurin-mediated dephosphorylation of the nuclear factor of an activated T cell (NFAT). Recent data indicate that Ca^2+^-calcineurin-NFAT1 to 4 pathways are dysregulated in autoimmune diseases. Therefore, calcineurin inhibitors, cyclosporine and tacrolimus, have been used for the treatment of such autoimmune diseases as systemic lupus erythematosus and rheumatoid arthritis. Here, we review the role of the Ca^2+^-calcineurin–NFAT signaling pathway in health and diseases, focusing on the STIM and Orai1, and discuss the deregulated calcium-mediated calcineurin-NFAT pathway in autoimmune diseases.

## Introduction

Calcium (Ca^2+^) is a second messenger that performs various functions, including cell division, activation, proliferation, and apoptosis in many cells (1) (Table 1). Ca^2+^ levels in the extracellular fluid (ECF) and endoplasmic reticulum (ER) lumen are maintained in the several millimolar (mM) range. However, the cytosolic Ca^2+^ levels are approximately in the range of 100 nM, which is 10,000 times lower than ECF Ca^2+^ concentrations (2). The resulting Ca^2+^ gradient is kept by intracellular Ca^2+^ stores, various types of Ca^2+^ channels, Ca^2+^/H^+^ ATPase, and Na+/Ca^2+^ exchangers. The cytosolic Ca^2+^ level is low in resting cells, but Ca^2+^-mobilizing agonists, such as hormones and growth factors, induce changes in the intracellular Ca^2+^ current dynamics. Cytosolic Ca^2+^ concentrations are balanced by the influx and efflux of Ca^2+^. There are two major routes of cytosolic Ca^2+^ influx: (1) from the intracellular stores, such as ER and mitochondria and (2) from the ECF. Ca^2+^ entry from the ECF is needed for the sustained elevation of cytosolic Ca^2+^ levels and full activation of Ca^2+^-dependent processes (1). On the other hand, emissions of Ca^2+^ occur through Na^+^/Ca^2+^ exchangers and plasma membrane Ca^2+^-adenylpyrophosphatase (ATPase).

**Table 1:** Role of elevated intracellular calcium (Ca^2+^) levels in various cells.

**Cell type**	**Effects**
Endothelial cells	Increase vasodilation
Secretory cells	Increase secretion, stimulate vesicle fusion
Juxtaglomerular cells	Decrease secretion
Parathyroid chief cells	Decrease secretion
Neurons	Stimulate transmission, vesicle fusion, and increase neural adaptation
Myocytes	Increase contraction and activation of protein kinase C
Keratinocytes	Stimulate differentiation
Lymphocytes	Stimulate T cells: Activation, anergy, motility, synapse formation, cytotoxicity, development, differentiation, and gene expression Stimulate B cells: Activation and maturation
Mast cells	Stimulate degranulation and histamine release
NK cells	Increase cytolytic activity in response to target cell recognition
Macrophage	Increase gene expression of pro-inflammatory cytokine, iNOS, and TNF
Dendritic cells	Stimulate maturation, migration of immature dendritic cells to secondary lymphoid organs Increase expression of MHC class II and co-stimulatory molecules
Neutrophils Osteoclasts	Increase phagocytosis, production of reactive oxygen species, degranulation, cytoskeletal rearrangement, and migration Osteoclast activation, differentiation, and survival

*NK, natural killer; iNOS, inducible nitric oxide synthase; TNF, tumor necrosis factor; MHC, major histocompatibility complex*.

Various Ca^2+^-permeable channels are involved in the influx of extracellular Ca^2+^: voltage-operated Ca^2+^ channels (VOCCs), receptor-operated channels (ROCs), store-operated channels (SOCs) like Ca^2+^ release-activated Ca^2+^ (CRAC) channels, and second messenger-operated channels (SMOCs) (1). VOCCs and ROCs are mainly located in electrically excitable cells, while SMOCs are in some excitable and non-excitable cells. In nerve and muscle fibers, electrically excitable cells, VOCCs and ROCs are the principal routes of Ca^2+^ entry. ROCs open in a few milliseconds when a neurotransmitter binds to them (3). SOCs are activated by second messenger molecules, usually inositol phosphates (IPs), diacylglycerol (DAG), and arachidonic acid and its metabolites (4). SOCs are found in all eukaryotes. CRAC channels are the main routes of Ca^2+^ entry in non-excitable cells, especially immune cells. When intracellular Ca^2+^ stores are decreased, store-operated Ca^2+^ entry (SOCE) is mediated by activated CRAC channels (1).

The intensity and length of Ca^2+^ signaling generated by SOCE and CRAC channels play different roles in immune cells. Short-duration functions occur within minutes. Short-duration types of Ca^2+^ signal transduction are not related to new gene expression, but to motility and the degranulation of T cells (5–7). In contrast, long-duration types of Ca^2+^ signaling sets to work on activation if the cytoplasmic Ca^2+^ levels are at higher-than-basal concentrations for hours through continuous Ca^2+^ entry. The long-duration types are associated with lymphocyte proliferation, expression of activation-related genes, cytokine or chemokine production, differentiation of T cells, and anergy (8).

Signaling between the plasma membrane and ER, or between ER and mitochondria, is required for the regulation of a direct Ca^2+^ transfer (9). It has long been recognized that inositol triphosphate serves Ca^2+^ release from the intracellular stores through inositol-1,4,5-triphosphate (IP_3_) receptor activation (4). However, recent discoveries of two essential regulators of CRAC channel functions have brought some new perspectives of cell functions regulated by Ca^2+^. The first regulator is stromal interaction molecule 1 (STIM1), a Ca2^+^ sensor protein presented in the ER, which is responsible for CRAC channel activation. The second regulator is Orai1, one subunit of CRAC channels. This review focuses on the roles of Ca^2+^ and the signal pathway upstream and downstream of Ca^2+^ flow in the immune cells and its involvement in autoimmunity and immunologic diseases.

## Calcium Channels

### Voltage-Operated Ca^2+^ Channels

VOCCs are located in electrically excitable cells, and they are activated by membrane depolarization. Usually, VOCCs function as the major passages of Ca^2+^ influx to the cells. However, their physiological importance or function in immune cells remains unclear.

### Ligand-Gated, Transient Receptor Potential (TRP) Channels

Ligand-gated Ca^2+^ channels are mostly non-selective ion channels. Among them, TRP channels consist of six subfamilies according to the amino acid sequence: the TRPC (canonical), TRPV (vanilloid), TRPA (ankyrin), TRPM (melastatin), TRPML (mucolipin), and TRPP (polycystin) groups (10). The TRPC1 was cloned to be the first mammalian member of the TRPC channel (11). Since then, TRPC2 and TRPC7 have been found. TRP channels are expressed in many cells and are expected to perform various biological functions. Usually, TRP channels function as polymodal cell sensors, but they also contribute to Ca^2+^ homeostasis. First, TRP channels function as Ca^2+^ entry via the plasma membrane (12). Second, when activated, they cause cell depolarization, which is the driving force for Ca^2+^ entry, and generate changes in the intracellular Ca^2+^ concentration (10). Third, TRP channels are also found in the ER and mitochondria and function as intracellular Ca^2+^-release channels (13).

There are a number of studies demonstrating the association between TRP and CRAC channels. For example, ORAI1, a CRAC channel, interacts with TRPCs and acts as a regulatory subunit that confers STIM1-mediated store depletion sensitivity to TRP channels (14). STIM1 also binds to TRPC1, TRPC4, and TRPC5 and is involved in SOCE (14). In unstimulated cells, TRPC1 does not display constitutive activation of the cells, and its levels in the plasma membrane are relatively low. However, TRPC1-containing vesicles can be found in the sub-plasma membrane region close to ER-plasma membrane junctions, in which Orai1 and STIM1 aggregate upon ER Ca^2+^ store depletion (15). This proximity enables TRPC1-containing vesicles to detect Ca^2+^ signals, the detection of which induces their recruitment to the plasma membrane (9). In the case of STIM1 knockdown, endogenous TRPC1-mediated SOCE and Ca^2+^ flow are significantly decreased (16). In contrast, exogenous co-expression of STIM1 with TRPC1 increases SOCE (16). If the store is sufficiently filled, TRPC1 is separated from STIM1 and TRPC1 is inactivated (16, 17). Activation of TRPC1 also relies on the presence of functional Orai1 since knockdown of Orai1 induces the complete elimination of TRPC1-mediated SOCE (14). Together, the previous studies suggest that plasma membrane expression of TRPC1 controls the Ca^2+^ signals in the cells in concert with STIM1 and Orai1. Further details regarding STIM1 and Orai1 are discussed below.

## Ca^2+^ Release-Activated Ca^2+^ Channels

### Stromal Interaction Molecule 1

Before the discovery of STIM proteins through the large-scale RNA interference (RNAi) screen (18), there were at least three models to regulate SOCE: conformational coupling (19), soluble Ca^2+^ influx factor (20), and vesicle fusion (21). However, these models could not fully explain the SOCE mechanism. STIM was identified through two RNAi screens carried out in *Drosophila* and mammalian cells (22). Unlike *Drosophila*, mammals express two STIM proteins, STIM1 and STIM2 (23). Both STIM proteins are single-pass transmembrane proteins with paired N terminal exchange factor (EF) hands located in the ER lumen (1). Protein interaction domains of STIM are found in both the ER lumen and the cytoplasm, while the Ca^2+^ binding EF-hand motif is placed in the portion of STIM1 facing the ER lumen (8). Mutations of Ca^2+^-binding glutamate and aspartate residues activate SOCE and CRAC channels independent of the filling state of the ER Ca^2+^ stores (18, 24), suggesting that STIM proteins serve as sensors of Ca^2+^ levels in the ER.

Both STIM1 and STIM2 can detect depletion Ca^2+^ stores in ER and lead to the activation of SOCE via Orai1. However, RNAi-mediated knockdown of STIM2 selectively decreases baseline levels of Ca^2+^ cytosolic and ER Ca^2+^ concentrations. Oh-Hora et al. (25) found that T cells and fibroblasts with a conditional deletion of STIM1 could result in a significant reduction of SOCE and CRAC channel function in C57BL/6 mice, which could be restored sufficiently by STIM1, but not recovered sufficiently by STIM2. The authors demonstrated that STIM1-deficient T cells showed only a transient nuclear localization of NFAT, whereas STIM2-deficient T cells demonstrated a normal, although unsustained, initial phase of nuclear localization of NFAT (25). Moreover, naive T cells lacking STIM2 performed normal SOCE and cytokine production (25). They concluded that STIM1, rather than STIM2, is the major functional Ca^2+^ sensor in ER that can trigger SOCE through CRAC channels in activated immune cells.

After Ca^2+^ store depletion, the relocation and aggregation of STIM1 forms small clusters (“puncta”) in the ER membrane (26). When ER Ca^2+^ has depleted, STIM1 starts dimerization or oligomerization and then forms plasma membrane clusters. STIM1 forms homo-multimers and hetero-multimers with itself and STIM2 (26, 27). These protein–protein interactions are mediated by protein interaction domains of STIM1, including a sterile α-motif (SAM), a coiled-coil ezrin, radixin, and moesin (ERM) domain, a serine- and proline-rich region, and a lysine-rich region (28). The location of puncta in or near the plasma membrane suggests that STIM1 may interact with CRAC channels (1, 24). Indeed, STIM1 puncta co-localizes with the lesion of Ca^2+^ entry and the CRAC channel subunit Orai1 (29). In contrast to STIM1, which can trigger SOCE, STIM2 seems to be involved in stabilizing basal cytosolic and ER Ca^2+^ levels (30). Parvez et al. (31) and Bema-Erro et al. (32) have reported that overexpressing STIM2 in cultured HEK293 cells increases resting intracellular Ca^2+^ levels, while cortical neurons, lacking STIM2, decreases them.

### Orai1

Genome-wide RNAi screens in *Drosophila* S2 cells revealed that depletion of the *olf186-F* gene (renamed *Drosophila* Orai) abrogates Ca^2+^ influx (33, 34). Again, RNAi-mediated knockdown of FLJ14466 (renamed ORAI1) (33), human homologs of *olf186-F*, interferes with CRAC channel functions and SOCE (34). *Orai1* is located on chromosome 12q24 in humans (33). Homozygosity for a missense mutation in *Orai1*, replacement of arginine with tryptophan at position 91 of the protein, abolishes the CRAC channel flow in T cells from patients with severe combined immunodeficiency (SCID) (8). Conversely, in T cells isolated from patients with SCID, overexpression of wild-type *Orai1* reconstitutes SOCE and CRAC channel flows (33). In this respect, Orai1 is considered to play a major role in the SOCE pathway.

**Figure 1:**
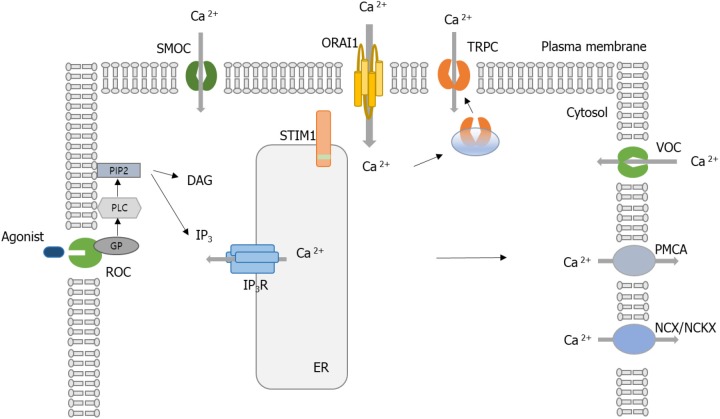
Schematic of calcium (Ca^2+^) regulation in a cell. Ca^2+^ entry is controlled by receptor-mediated Ca^2+^ (ROC) entry, transient receptor potential (TRP) channels, voltage-gated Ca^2+^ channels (VOCC), and Ca^2+^ release-activated Ca^2+^ (CRAC) activated by the STIM1 protein. Ca^2+^ efflux is mediated by plasma membrane (PM) Ca^2+^ ATPase (PMCA), Na^+^/Ca^2+^ exchanger (NCX), or Na^+^/Ca^2+^/K^+^ exchanger (NCKX). When the Ca^2+^-mobilizing agonist (e.g., receptor engagement by antigen) binds to ROC, it results in the activation of phospholipase C (PLC). PLC cleaves the phosphatidylinositol-4,5-bisphosphate (PIP_2_) to generate the following second messengers, inositol-1,4,5-triphosphate (IP_3_) and diacylglycerol (DAG). IP_3_ binds to IP_3_ receptors (IP_3_Rs) located on the surface of the endoplasmic reticulum (ER) and activates the release of Ca^2+^. The release of Ca^2+^ from the ER and sarcoplasmic reticulum (SR) occurs through IP_3_R. When ER Ca^2+^ stores are depleted, STIM1 aggregates to the ER–PM junction. STIM1 recruits the Orai1, and then the CRAC channel is activated. Influx of Ca^2+^ via Orai1 induces the recruitment of TRPC1 from vesicles into the PM. SMOCs, second messenger-operated channels; GP, G proteins.

When Orai1 was discovered, researchers wondered if Orai1 could be a component of the CRAC channel or a protein that relates the opening of CRAC channels. Several groups investigated the site-directed mutagenesis of conserved glutamates in the first and third predicted transmembrane domains of *Drosophila* and human Orai1 to show that both *Drosophila* and human Orai1 are elements of the CRAC channel pore (33–35). Human Orai1 shares 73% sequence homology with *Drosophila* Orai (36). Based on the *Drosophila* Orai structure, human Orai1 channels are expected to have a hexameric structure comprising three dimeric subunit pairs (36). The central aqueous pore of Orai1 is created from the six pore-forming N-terminal transmembrane helices (TM1). TM2 and TM3 surround TM1, while TM4 forms the periphery of the channel. Previous Orai1 mutagenesis studies have indicated that a set of conserved acidic amino acids in TM1 and TM3 and in the TM1-TM2 loop (E106, E190, D110, D112, D114) is essential for the Ca^2+^ selectivity filter of the CRAC channel (37). Prakriya et al. (35) replaced the corresponding glutamates (E106 and E190) in human *Orai1* with alanine (A), aspartate (D), or glutamine (Q). The mutant proteins were transduced into SCID T cells, and then SOCE was analyzed (35); as mentioned earlier, SCID disease is characterized by the absence of SOCE and CRAC channel currents (33). The authors found that mutations at E106 and E190 significantly decreased SOCE. Moreover, E106D and E190Q mutation greatly decreased Ca^2+^ selectivity of the CRAC channel (35). Similar observations were reported by a study using *Drosophila* Orai (38), where overexpression of *Drosophila* Orai in S2 cells resulted in a great increase in SOCE and CRAC currents (38). Another human study using overexpressed human Orai1 in HEK293 cells also confirmed that E106 and E190 are essential sites for CRAC channel function (39). Taken together, Orai1 has been considered a critical component of the pore of the CRAC channel. Figure 1 shows the above Ca^2+^ channels associated with Ca^2+^ homeostasis.

## Ca^2+^ Signaling in Lymphocytes

Among the various Ca^2+^ channels, CRAC channels function uniquely in immune cells, especially lymphocytes (1). CRAC channels are opened differently depending on a lengthy signaling cascade. Short-duration functions are generally established within minutes. They are involved in modulation of lymphocyte motility, and the degranulation of cytotoxic CD8^+^ T cells (5–7). The engagement of T cell receptor (TCR) or B cell receptor (BCR) with antigen-presenting cells (APCs) bearing antigenic peptides leads to a quick elevation of intracellular concentrations of Ca^2+^. As Ca^2+^ levels increase, T cells stop their movement and form stable immunological synapses. The long-duration functions include cell proliferation, expression of activation-related genes, production of cytokines/chemokines, and differentiation of lymphocytes (40). The continuous Ca^2+^ influx is required since this long-duration function occurs when Ca^2+^ concentration maintains higher-than-basal levels for several hours.

### Ca^2+^ Upstream Signaling in Lymphocytes

When antigen/major histocompatibility complex (MHC) complexes bind to TCR, protein tyrosine kinases are activated. This binding induces the tyrosine phosphorylation and the activation of phospholipase C, gamma 1 (PLC-γ1) (4). PLC-γ1 catalyzes the formation of IP_3_ and DAG from the membrane phospholipid phosphatidylinositol-4,5-bisphosphate (PIP_2_). IP_3_ opens IP_3_ receptors (IP_3_R) and allows Ca^2+^ efflux from the ER Ca^2+^ reservoir (4). STIM proteins recognize decreased Ca^2+^ concentration in the ER through their canonical EF hands located within the ER lumen, acting as Ca^2+^ sensors of the ER (1). When the Ca^2+^ is detached from the EF hands, STIM forms clusters in the ER membrane. This conformation contacts the Orai1 pore channel subunit, triggering SOCE (1).

### Ca^2+^ Downstream Signaling Pathway After Ca^2+^ Influx

Once Ca^2+^ influx increases intracellular Ca^2+^ concentration, intracellular Ca^2+^ activates other signaling pathway and transcription factors. The pathway known to date is (1) the calmodulin–calcineurin pathway, with the final activation of the NFAT1 to (2, 4) Ca^2+^-dependent kinase-calmodulin (CaMK), and (3) nuclear factor κB (NF-κB). Calmodulin is a Ca^2+^-binding messenger protein expressed in all eukaryotic cells. When Ca^2+^ binds to calmodulin, it forms the Ca^2+^-calmodulin complex (1). This complex activates Ca^2+^ pumps, which then removes Ca^2+^ from the cytoplasm or stores Ca^2+^ in the ER. The Ca^2+^-calmodulin complex also activates calmodulin-dependent kinase (CAMK), which phosphorylates several effector proteins by transferring phosphates from ATP to serine and threonine residues on the proteins (1). Calcineurin is a calmodulin-dependent serine/threonine phosphatase. Calcineurin is composed of a calmodulin-binding catalytic subunit, calcineurin A, and a regulatory subunit, calcineurin B (1).

In mammals, there are three isoforms of calcineurin A (calcineurin Aα, calcineurin Aβ, and calcineurin Aγ) and two isoforms of calcineurin B (calcineurin B1 and calcineurin B2) (40, 41). When intracellular Ca^2+^ concentration increases the calmodulin binding to calcineurin, NFAT translocates to the nucleus (40–42) and then upregulates transcription of its target genes that are essential for innate and adaptive immunity. All NFAT1 to NFAT4, except NFAT5, are activated by calcineurin. They have conserved the N-terminal domain containing calcineurin binding sites (43). Peripheral lymphocytes express NFAT1, NFAT2, and NFAT4. Single-positive thymocytes preferentially expressed NFAT1. NFAT2 is expressed in double-negative thymocytes and B cells, and NFAT4 is expressed in double-positive thymocytes (44, 45), suggesting that NFAT isoforms of NFAT play different roles at the stage of the development and in the maturation of T cells in the thymus. Such a notion is supported by a recent study showing that NFAT1 and NFAT4 require distinct subcellular InsP_3_ and Ca^2+^ signals for physiologically sustained activation (46).

## Ca^2+^ Signaling in Other Immune Cells

### Neutrophils

It is widely accepted that the Ca^2+^ signal is crucial to a variety of functions of neutrophils as well as lymphocytes. In neutrophils, Ca^2+^ signaling starts with the binding of various cell surface receptors, including Fc-gamma receptors (FcγRs), G-protein-coupled receptors (GPCRs), and integrins (47). Adhesion of neutrophils generates Ca^2+^ influx, which controls exocytic events associated with the movement of neutrophils (48). Increased Ca^2+^ generated by integrin engagement triggers a rapid change in neutrophil morphology and accelerates the neutrophil spreading (49). Intracellular Ca^2+^ also is involved in the assembly and disassembly of actin during neutrophil adhesion and phagocytosis (50, 51). Moreover, the Ca^2+^ signal seems to mediate the exocytic process as evidenced by studies demonstrating that the release of different granules depends on the Ca^2+^ concentration in neutrophils (52).

As expected, two CRAC channels, STIM1 and Orai1, have been implicated in neutrophil functions. For example, inhibition of STIM1-induced SOCE using siRNAs leads to a marked decrease in NADPH oxidase activity in neutrophil-like HL-60 cells, while STIM2 siRNA has no effect (53). STIM1 siRNAs also reduce the polarization of HL-60 cells possibly through the Akt/Src/Rac pathways (54). In addition, STIM1 interacts with Ca^2+^ channels on phagosomes to promote localized Ca^2+^ elevations that drive high-efficiency phagocytosis in neutrophils (55). Moreover, knockdown of Orai1 using siRNA reduces SOCE and ROS production in HL-60 cells (56, 57), suggesting that Orai1 also mediates the SOCE in neutrophils. Collectively, it seems likely that Ca^2+^ flux mediated by STIM1 and Orai1 is required for adequate functions of neutrophils, including phagocytosis.

Unlike phagocytosis, there is a discrepancy whether STIM1 regulates neutrophil migration. In the psoriatic inflammation animal model, conditional knockout mice lacking STIM1 have less neutrophil infiltration in the epidermis than controls (58), indicating that STIM1 is essential for chemotaxis. In a sharp contrast, Zhang et al. (59) have reported that neutrophils of *stim1*^−/−^ mice display no defect in adhesion and migration. Moreover, in a human study of patients with loss-of-function mutations in ORAI1 and STIM1 genes, Ca^2+^ influx is only modestly reduced in ORAI1- and STIM1-deficient neutrophils (60). Moreover, antibacterial cellular functions, including phagocytosis, adhesion, and chemotaxis, are preserved in human neutrophils mutated in ORAI1 and STIM1 genes (60). Therefore, it is not conclusive that STIM1 and ORAI1 are actually so critical for effector functions of neutrophils. There might be another possibility that other Ca^2+^ channels or membrane proteins mediate SOCE and effector functions of human neutrophils. Further research will be required to clarify this issue.

### Osteoclasts

It is well-known that signaling by the receptor activator of nuclear factor-κB ligand (RANKL) critically regulates the differentiation of monocytes/macrophages to osteoclasts, the key immune cells involved in bone resorption and destruction. An increasing body of evidence suggests that the Ca^2+^-calcineurin–NFAT pathway is also essential for the activation and differentiation of osteoclasts. A decrease in cytosolic Ca^2+^ by extracellular protons promotes the expression of cell matrix attachment structures (61). The Ca^2+^ sensing receptor is also directly involved in osteoclast differentiation and apoptosis (62). Interestingly, RANKL evokes Ca^2+^ oscillation, then leads to calcineurin-mediated activation of NFATc1, and promotes osteoclast differentiation in NFATc1-dependent manners. Yang and Li (63) also have demonstrated that RANKL sequentially induces Ca^2+^ oscillation, NFATc1 activation, and osteoclast differentiation, which is almost completely dependent on the regulator of G-protein signaling 10, a regulatory molecule that acts as GTPase activating proteins, which suggests that the Ca^2+^-dependent NFAT pathway is the key downstream signaling pathway of RANKL for osteoclast differentiation.

Like in lymphocytes and neutrophils, there are studies showing that STIM1 and Orai1 are required for osteoclastogenesis. STIM1 is highly expressed in osteoclasts at an early stage (64). *Stim1* and *Orai1* silencing inhibits RANKL-induced Ca^2+^ oscillation in RAW264.7 macrophages (65). Moreover, knockdown of Orai1 suppresses multi-nucleation of osteoclast precursor cells and consequently inhibits osteoclastogenesis of RAW264.7 cells (66). Besides STIM1 and Orai1, TRP channels also are expressed in osteoclasts (67) and contribute to osteoclastogenesis (68). Knockdown of TRP5 in human osteoclasts reduces RANKL-induced Ca^2+^ influx (69). In an experiment using TRPV4^−/−^ mice, it has been demonstrated that TRPV4 regulates Ca^2+^ signaling, activates NFATc1, and enhances the differentiation and survival of osteoclasts (70).

## Association OF Ca^2+^-Calcineurin–NFAT Axis With Immunologic Diseases

These autoimmune diseases are characterized by the presence of autoreactive T cells that lead to the generation of the inflammatory process. Intracellular Ca^2+^ signaling in T cells has been implicated in the pathogenesis of autoimmune disease (1). In the thymus, immature T cells develop from progenitors through positive and negative selection. Even when autoreactive T cells happen to have escaped from such selection in the thymus, they can be suppressed by regulatory T cells (Tregs) to maintain peripheral tolerance. Of note, the Ca^2+^ signal is thought to be an important modulator of TCR signaling strength as well as a regulator of T cell development and selection (1). In fact, Ca^2+^ oscillations are found under positively selecting conditions in thymic slices (1, 71, 72). Strong Ca^2+^ influx is associated with negative selection of T cells induced by high-affinity peptide ligands, but moderate Ca^2+^ influx is related to positive selection by weak peptide–MHC–TCR interactions (73). Recently, patients with a mutation of STIM1 have been reported (74). They develop autoimmune diseases due to a severe reduction in peripheral Foxp3^+^ Treg cells. Meanwhile, after encountering APCs loaded with the appropriate peptide, naive Th cells differentiate into specific Th cell subsets, including Th1, Th2, and Th17, depending on the local cytokine milieu. In such a polarization step, intracellular Ca^2+^ levels can be measured differently according to Th subtype. For example, intracellular Ca^2+^ concentrations in resting state are higher in Th2 cells than in Th1 cells and intermediate in Th17 cells (75), which suggests that a defect of Ca^2+^ signaling can affect Th cell differentiation and polarization. In this section, we describe the functional role of Ca^2+^ signaling in the pathogenesis of autoimmune diseases, including rheumatoid arthritis (RA), systemic lupus erythematosus (SLE), Sjögren's syndrome (SS), and psoriasis, focusing on dysregulated Ca^2+^ signaling in autoreactive T cells (Table 2).

**Table 2:** Association of calcium (Ca^2+^) channels and various autoimmune diseases.

**Disease**	**Channels and Cells**	**Effects**
Rheumatoid arthritis	Upregulation of CRAC on T cells Upregulation of TRPV1 and TRPM8 on synoviocytes Activation of TRPV4 on synoviocytes Activation of TRPV2 on synoviocytes	Increase Ca^2+^ influx and NFAT transcription→ stimulation of secretion of inflammatory cytokines Activate caspase enzyme→ induce apoptosis of FLS Increase Ca^2+^ influx reduce→ chemokine production Increase intracellular Ca^2+^ concentration→ reduce joint inflammation
Systemic lupus erythematosus	Altered motility of Kv1.3 on T cells	Activate T cell proliferation and T cell-mediated autoimmune responses
Sjogren syndrome	Downregulation of CRAC on T regs Inhibition of TRPC3 on salivary gland	Reduce SOCE and Treg functions→ decrease salivary gland secretion Reduce Ca^2+^ influx→ reduce cell-mediated toxicity→ decrease inflammation
Psoriasis	Downregulation of TRPV6 on keratinocytes Downregulation of STIM1	Reduce Ca^2+^ influx→ inhibit keratinocyte differentiation and proliferation Reduce Ca^2+^ influx→ decrease chemotaxis of neutrophils
Multiple sclerosis	Upregulation of CRAC on T cells	Change Ca^2+^ entry Stimulate T cell activation and proliferation→ increase cytokine production

*CRAC, Ca^2+^ release-activated Ca^2+^; NFAT, nuclear factor of activated T cell; TRPV, transient receptor potential vanilloid; TRPM, transient receptor potential melastatin; FLS, fibroblast-like synoviocyte; SOCE, store-operated Ca^2+^ entry; Tregs, regulatory T cells; TRPC, transient receptor potential canonical*.

### Rheumatoid Arthritis

RA is a chronic systemic inflammatory disease. Pathologically, chronic inflammation in the synovia spreads to the surrounding cartilage and bone, and if left untreated, causes joint destruction, deformity, and disability. Genetic and environmental factors, including human leukocyte antigen (HLA) DR1 and 4 subtypes, smoking, and infectious agents, are thought to contribute to RA etiology and pathogenesis (76). Most RA patients have autoantibodies to self-antigens, which include the rheumatoid factor (RF) and anti-cyclic citrullinated peptide antibodies (ACPA), indicating that humoral immunity is involved in disease pathogenesis (77). There is also ample evidence of the presence of autoreactive T cells in the synovium (78), the association of autoreactive T cells with HLA-DR1/4 and T cell-derived cytokines, including interferon gamma (IFN-γ) and interleukin-17 (IL-17) (78), and the clinical efficacy of anti-T cell therapy in RA patients (79). When chronically exposed to various cytokines and growth factors derived from activated lymphocytes and other types of immune cells, resident synoviocytes are activated, proliferated abnormally, and de-differentiated to an aggressive phenotype, reminiscent of cancer cells (80).

Although CD4^+^ T cells are thought to be the principal mediators of RA progression, how CD4^+^ T cells are induced and activated needs clarification. Sakaguchi et al. (81) reported that spontaneous point mutation of the gene encoding an SH2 domain of ZAP-70, an important signal transduction molecule in T cells, leads to chronic autoimmune arthritis similar to RA in mice. In this animal model, TCR signals, including Ca^2+^ signaling, are markedly impaired (81). Altered signal transduction from TCR through the ZAP-70 mutation changes the thresholds of T cells for thymic selection, leading to the positive selection of otherwise negatively selected autoimmune T cells. Together, those findings imply that Ca^2+^ signals are involved in fine-regulating TCR signals in thymocytes, and accordingly in the selection of functionally healthy T cells, and the depletion of autoreactive T cells.

Since RA has a strong genetic background, there is research into whether it is associated with Ca^2+^ regulatory genes. Yen et al. (82) investigated 400 RA patients and 621 healthy controls for a case-control genetic association study to find whether *Orai1* is involved in RA susceptibility. Five tagging single-nucleotide polymorphisms (SNPs) within *Orai1* were selected for genotyping. As a result, the SNP rs7135617 has a significant correlation with the risk of RA, indicating that genetic polymorphism of *Orai1* contributes to the susceptibility to RA.

Because Ca^2+^ is a key regulator of a variety of transcription factors, control of Ca^2+^ influx is essential for the activation and function of the adaptive immune response. Liu et al. (83) studied whether CRAC channels contribute to the abnormal behavior of T cells in RA. They found a significant positive correlation between Ca^2+^ influx in naive T cells and RA activity. Moreover, functionally aberrant naive CD4^+^ T cells from active RA patients were found to show different cytokine release patterns, enhanced Ca^2+^ influx, and increased expression and function of CRACM1, a CRAC protein (83).

As a downstream target of Ca^2+^, the calcineurin–NFAT pathway is required for RA pathogenesis. NFAT is expressed in RA synovium at high levels (80). The presence of calcineurin has also been demonstrated in monocytes/macrophages and vascular endothelial cells in RA synovium (84). Moreover, calcineurin upregulates the expression of pathogenic T cell-derived cytokines, including IL-17 and tumor necrosis factor-α (TNF-α), in RA (84). Beyond its role in T cells, calcineurin induces the activation of synoviocytes and chondrocytes (85, 86). We have reported that calcineurin expression is higher in the synoviocytes of RA patients than in those of osteoarthritis patients (86). Pro-inflammatory cytokines increase calcineurin activity in synoviocytes, and in turn, increased calcineurin activity triggers the production of IL-6 and matrix metalloproteinases (MMPs). Ca^2+^ stores in the non-ER compartment are increased in the synoviocytes of RA patients, which underlies the altered Ca^2+^ signaling in RA, thus making these cells hyperresponsive to external stimuli, including TNF-α. Calcineurin is also expressed in chondrocytes. The inhibition of calcineurin decreases IL-1β, MMP1, and MMP3 production while increasing type II collagen, tissue inhibitor of metalloproteinases-1, and transforming growth factor-β (TGF-β) expressions, suggesting that calcineurin regulates the catabolic and anabolic activities of chondrocytes (85).

**Figure 2:**
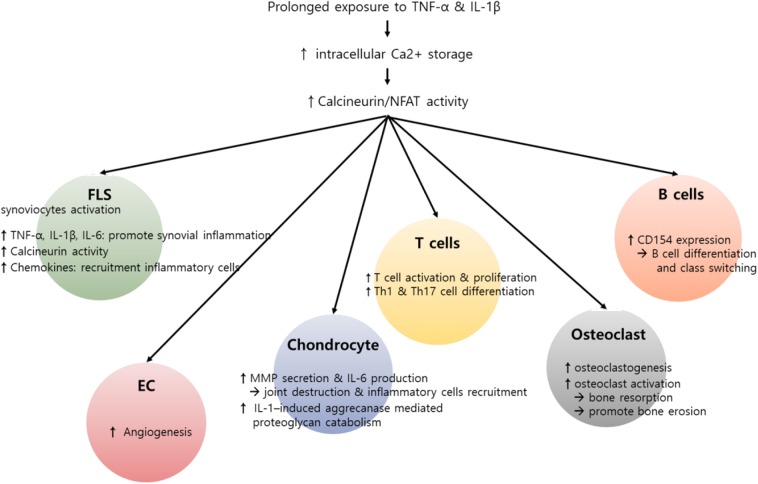
Potential role of calcium (Ca^2+^)–calcineurin–nuclear factor of an activated T cell (NFAT) pathway in innate and adaptive immune cells involving rheumatoid arthritis (RA) pathogenesis. RA is characterized by infiltration of various inflammatory cells such as macrophages, T cells, and B cells, in addition to hyperactivation and proliferation of fibroblast-like synoviocytes (FLSs). Pro-inflammatory cytokines, including interleukin-1β (IL-1β) and tumor necrosis factor-α (TNF-α), can induce an increase in intracellular Ca^2+^ concentration. Subsequently, activated calcineurin propagates a wide range of signals essential to the hyperactivation of diverse immune cells involving RA pathogenesis. In T cells, activated calcineurin promotes NFAT and nuclear factor κB (NF-κB) and decisively controls T cell function, growth, and apoptosis. The calcineurin–NFAT axis can also upegulate CD154 transcription, thereby inducing B cell differentiation and antibody production. Beyond its role in lymphocytes, Ca^2+^-calcineurin–NFAT seems to directly activate innate and matrix metalloproteinases. Calcineurin–NFAT signaling mediates vascular endothelial growth factor (VEGF)-induced endothelial migration and proliferation and thereby can enhance pathologic angiogenesis in RA synovia (88, 89). Additionally, the receptor activator of nuclear factor-κB ligand (RANKL)-induced increase in intracellular Ca^2+^ levels triggers the Ca^2+^-calcineurin pathway in osteoclasts, which promotes osteoclastogenesis through NFATc1 activation. Taken together, dysregulated intracellular Ca^2+^ store and Ca^2+^ response contribute to the pathogenesis of RA by activating calcineurin–NFAT pathway in multiple types of cells.

Taken together, the Ca^2+^-calcineurin–NFAT axis is upregulated in various types of synovial inflammatory cells of RA patients, promotes the production of pro-inflammatory cytokines and MMPs, and thereby plays an important role in maintaining chronic inflammation in RA. In fact, calcineurin inhibitors, including cyclosporine and tacrolimus, have been widely used over recent decades with great efficacy in the treatment of RA (87). We believe that the action of such inhibitors is not limited to T cells but also targets non-T cells, including macrophages and synoviocytes, and therefore the therapeutic efficacy of calcineurin inhibitors in RA comes from their combinatory action on multiple types of pathologic immune cells. Figure 2 summarizes the role of the Ca^2+^-calcineurin–NFAT pathway in RA pathogenesis.

### Systemic Lupus Erythematosus

SLE is a systemic autoimmune disorder involved in multiple organs, leading to tissue damage to any part of the body with diverse clinical manifestations. The pathologic findings of SLE occur through inflammation, blood vessel abnormality, and immune complex deposition. In SLE, the major immunologic disturbance is autoantibody production (e.g., anti-double-strand DNA, anti-Sm, anti-ribonucleoprotein, anti-SSA, and anti-SSB), which is caused by a generalized immune cell dysfunction that promotes B cell hyperactivity. Several studies have demonstrated abnormal signaling through BCR, and this modified signaling results in increased Ca^2+^ signals (90–94). It has been reported that B cell stimulation in SLE patients with antibodies against surface IgM shows an elevated intracellular Ca^2+^ response compared to healthy controls (90). Such increased Ca^2+^ entry was found to be associated with impaired FcγRIIB signaling and reduced expression levels of Src-homology 2-domain phosphatase-1 (SHIP-1) (91).

Physiologically, FcγRIIB diminishes Ca^2+^ signals via the activation of SHIP and hydrolysis of phosphatidyl-inositol-3,4,5-trisphosphate and also by inhibiting CD19-mediated Ca^2+^ responses (92). Therefore, a point mutation in the murine *phospholipase C*γ*2 (PLC*γ*2)* can lead to severe spontaneous inflammation and systemic autoimmunity (94). This underlying mechanism is a gain-of-function mutation in *PLC*γ*2*, resulting in increased Ca^2+^ responses in B cells and the expansion of innate inflammatory cells. Such mutation leading to autoimmunity is also noted in another animal model that is deficient of inhibitors of BCR signaling, including SHP1 and LYN, where Ca^2+^ responses in B cells increase and, in parallel, autoimmune symptoms resembling SLE develop (93). Similarly, it has been documented that B cells from CD22-deficient mice lead to elevated Ca^2+^ influx on BCR ligation and autoantibody production (95). Taken together, the aforementioned reports support the notion that abnormal B cell signaling linked to the increased Ca^2+^ responses can break B cell tolerance down and induce autoimmunity and autoimmune symptoms related to SLE.

It is well-known that T cells inappropriately help B cells to produce pathogenic autoantibodies. Recent studies on the detailed mechanisms of T cell differentiation have provided better understanding of the more complicated role of T cells in the pathogenesis of SLE (96–100). In particular, Th1, Th17, and follicular helper T cells are activated and expanded, whereas Tregs are dysfunctional in SLE patients (96), and such imbalance in Th cell subtypes is believed to play a major role in SLE pathogenesis (97). Interestingly, nuclear NFAT levels are abnormally elevated in activated T cells from patients with SLE (98), although which type of Th cells shows such abnormality in SLE remains elusive. Once SLE T cells are activated through their TCR, Ca^2+^ influx increases, which induces calcineurin-mediated NFAT translocation to the nuclei, and then promotes transcriptional upregulation of CD40 ligand, a co-stimulatory molecule that induces antibody production and dendritic cell activation (99). Since CD40–CD154 signaling induces the differentiation of T cells into Th17 subtype (98, 100), it can be postulated that the increase in NFAT signaling may contribute to the Th17 polarization noted in SLE by upregulating the CD40 ligand expression.

Another example of abnormalities in the Ca^2+^-calcineurin pathway in SLE would be with CD4^−^CD8^−^ (double-negative) T cells. These double-negative T cells produce more inflammatory cytokines, including IL-17 and IFN-γ (101). They are expanded in SLE patients and can directly invade such diverse organs as the kidneys and the skin. Intriguingly, double-negative T cells are characterized by early and robust Ca^2+^ influx, and these cells help B cells in a calcineurin-dependent manner (102), raising the possibility that the Ca^2+^ signal contributes to the SLE pathogenesis by promoting the activity of double-negative T cells.

Taken together, the aforementioned studies suggest that the Ca^2+^-calcineurin–NFAT axis is abnormally activated in the T and B cells of SLE patients, breaks B cell tolerance, and induces T cell differentiation toward pathogenic Th17 subtype, and so can thus be a therapeutic target of SLE. In fact, dipyridamole, which is accompanied by a decrease in the frequency of the double-negative T cells (101), inhibits the calcineurin–NFAT pathway, suppresses the production of pro-inflammatory cytokines and co-stimulatory molecules by T cells, and alleviates lupus nephritis and skin ulcers. In parallel, cyclosporine A and tacrolimus, calcineurin inhibitors, show a significant renal protective effect and have been widely used in SLE patients (103, 104).

### Sjögren's Syndrome

SS is a systemic autoimmune disease caused by the lymphocytic infiltration of T cells into exocrine glands, including lacrimal and salivary glands, leading to the destruction of the glands. Pathologically, the earliest lymphocytic infiltrates are composed of T cells, mostly of the CD45RO primed memory Th phenotype and CD20^+^ B cells (105). The majority of patients with SS complain of dry eye and xerostomia. The extra-glandular features of SS include arthralgia, thyroid disease, peripheral neuropathy, renal involvement (e.g., renal tubular acidosis), and cutaneous vasculitis. Most patients with SS show an increased frequency of circulating autoantibodies, including two specific antibodies directed against the Ro (SS-A) and La (SS-B) antigen.

Several studies in animal models have unveiled the potential involvement of STIM1 and STIM2 in SS pathogenesis (24, 106). STIM1 and STIM2 knockout mice exhibit decreased salivary gland secretion, decreased Ca^2+^ entry, and dysfunction of Tregs, but they also show elevated levels of salivary gland-specific antibodies (106), which demonstrate a possible link between STIM1/2 deficiency and T cell dysfunction associated with SS pathogenesis. Moreover, in an animal model with T lymphocyte-targeted deletion of STIM1 and STIM2, SOCE and SOCE-dependent cytokine productions are significantly decreased, and the number and function of Tregs are substantially reduced (24). Those mice display signs of autoimmunity, including increased infiltration of lymphocytes into glandular tissues, suggesting that the loss of STIM proteins and subsequent impairment of SOCE in T lymphocytes lead to defects in Treg function and autoimmune glandular destruction (106). Of note, STIM1 protein levels, STIM2 protein levels, and SOCE function are all decreased in peripheral blood mononuclear cells from SS patients (106). In aggregate, the previous studies suggest that the dysfunction of STIM proteins could be the basis for the onset and progression of SS. In accordance with this, there are some pilot studies that demonstrate that the calcineurin inhibitor, cyclosporine A, is effective in SS patients with ocular symptoms or articular involvement (107).

### Psoriasis

Psoriasis is a chronic inflammatory skin disease characterized by various sized thick scaly erythematous plaques (108). The histopathology of psoriatic plaques shows epidermal proliferation and inflammation of the dermis (109). Both innate and adaptive immune cells, including keratinocytes and T cells, participate in the initiation and perpetuation of psoriasis (58, 110). Psoriasis is a well-established T cell-mediated skin disease (110, 111). In particular, various cytokines induce the activation of immune cells, particular Th1 and Th17 cells (111), and the functional imbalance of Th1 or Th17 over Tregs is considered a key pathway for the progression of psoriasis (111). For example, psoriatic skin lesions show a strong IFN-γ signature and have an abundance of IFN-γ (+) Th1 cells (112). An imbalance between Tregs and effector T (Teff) cells is observed in the peripheral blood of psoriasis patients (113). Moreover, the Tregs of psoriasis patients are functionally deficient in suppressing Teff cells (114). Recently, the association between IL-9 and the Th17 pathway has been reported in psoriasis. Expressions of IL-9 and IL-9R are markedly increased in psoriatic skin lesions (115), and IL-9 stimulates the production of IL-17A by CD4^+^ T cells isolated from patients with psoriasis (116).

It is well-established that calcineurin inhibitors suppress T cell activation and the differentiation of naive T cells to memory T cells (117). In particular, calcineurin inhibitors downregulate the expression of *STAT1, IFN*-γ, and several *IFN*-γ-downstream genes, repressing the generation of Th1 cells (118). Moreover, the expressions of *IL-17, IL-22*, and *IL-17*-inducible genes, including *DEFB-2, LCN2, IL-1*β, *S100A12*, and *CCL20*, are markedly suppressed by calcineurin inhibitors (119). Given the importance of Th1 and Th17 cells in psoriasis pathogenesis, the inhibition of the calcineurin–NFAT pathway seems to be therapeutically relevant to psoriasis. Interestingly, the actions of calcineurin inhibitors are not limited to T cells. NFAT1 expression was first described by Northrop et al. (120) in mice skin, and then calcineurin expression was subsequently reported in the human epidermis (121). Calcineurin inhibitors reduce antigen presentation by Langerhans' cells and suppress neutrophil chemotaxis through the inhibition of psoriatic monocytes (122). Epidermal IL-1 and IL-8 expressions in psoriatic skin can be blocked by the calcineurin inhibitor cyclosporine (123). Indeed, cyclosporine and tacrolimus, both calcineurin inhibitors, have been widely used in psoriasis treatment with high efficacy (124).

STIM1 and Orai1 in keratinocytes, CRAC channels, have been implicated in the proliferation and differentiation of keratinocytes. It has been demonstrated that keratinocyte differentiation is induced by the change of extracellular Ca^2+^ concentration (125). Increased extracellular Ca^2+^ concentration triggers phospholipase C-mediated intracellular Ca^2+^ signals, which activate SOCE. Moreover, siRNA-mediated knockdown of either STIM1 or Orai1 suppresses SOCE and almost completely abolishes the Ca^2+^-mediated keratinocyte differentiation and growth (125). Menon and Elias (126) reported a defective Ca^2+^ gradient in the keratinocytes of psoriasis patients. Keratinocytes isolated from psoriasis patients showed a decreased response after Ca^2+^ store depletion as well as reduced mRNA/protein expression of CRAC channels (127, 128). In line with these findings, another study reported reduced mRNA and protein expression of TRPC channels (128), and the incubation of keratinocytes isolated from psoriasis patients with the TRPC6 agonist partly restores their differentiation and proliferation defect (129). Therefore, it remains to be determined whether Ca^2+^ sensing and signaling pathway plays an inductive or protective role in the pathologic differentiation and proliferation of keratinocytes in psoriasis.

Taken together, the earlier reports indicate that Ca^2+^ sensing and signaling pathway can be an excellent target for the treatment of psoriasis. Currently, phase 1 of a clinical trial of CRAC channel inhibitor for plaque psoriasis is in progress. CRAC channel inhibitors, a new class of oral immunomodulatory drugs, potently inhibit Orai1, Th1, Th2, and Th17-derived cytokine production and T cell proliferation, which are involved in chronic inflammatory responses in psoriasis (130).

### Experimental Autoimmune Encephalomyelitis (EAE)

Experimental autoimmune encephalomyelitis (EAE) is an animal model of brain inflammation, where autoreactive T and B cells to neuro-antigens, such as myelin basic protein and myelin oligodendrocyte glycoprotein (MOG), play a major role. EAE is a representative Th1 and Th17 cell-mediated central nervous system (CNS) disease (131) and has been widely used as an animal model of human demyelinating diseases, including multiple sclerosis and acute disseminated encephalomyelitis.

There are several studies demonstrating that dysregulated Ca^2+^ signal pathway is closely related to the development of EAE. For example, decreased levels of STIM1 protein greatly impair the production of neuro-antigen-specific T cell responses (reduced Th1/Th17 responses), and this results in complete protection from EAE (131). STIM2-deficient mice develop EAE, but the severity of disease is mild. Deficiency of STIM2 was thought to be associated with the hypo-proliferation of lymphocytes and a reduction of IFN-γ/IL-17 production by neuro-antigen-specific T cells (132). In accordance with this, immunization of mice deficient in Orai1 in T cells with MOG peptide yields to improvement of EAE severity. These mice are shown to have almost completely suppressed the production of IL-17A, IFN-γ, and granulocyte macrophage-colony stimulating factor (GM-CSF) (133), suggesting that CRAC channels, including STIM and Orai1, modulate Th1 and Th17 responses and could therefore be a therapeutic target of EAE.

Interestingly, SOC influx induced by STIM1 and STIM2 is important to B cell regulatory function (134). B cell-specific deletion of STIM1 and STIM2 in mice causes a significant defect in BCR-induced Ca^2+^ entry and B cell proliferation and fails to produce IL-10 because of the defective activation of NFAT after BCR stimulation, resulting in the exacerbation of EAE. It therefore seems likely that STIM-dependent SOC influx is the major signal for Th cell differentiation and B cell activation, but the net *in vivo* effect of blockades of STIM1/2 activity in immune cells on EAE development and autoimmunity remains elusive.

## Conclusion and Future Perspectives

**Figure 3:**
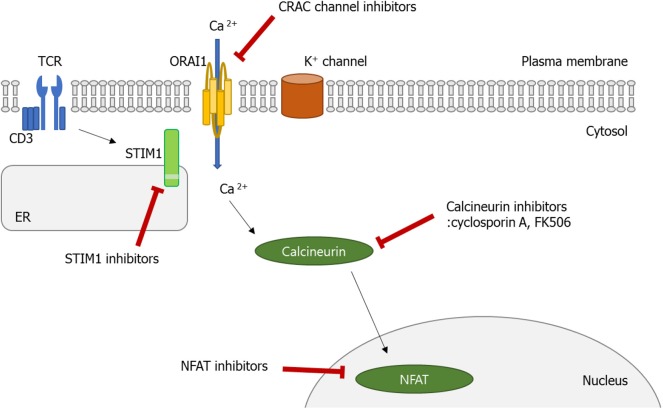
Calcium (Ca^2+^)–calcineurin–nuclear factor of an activated T cell (NFAT) signaling pathway as a novel therapeutic target. Orai1 is a plasma membrane protein with four transmembrane segments. Stromal interaction molecule 1 (STIM1) is a single-pass transmembrane protein located in the endoplasmic reticulum (ER). The increase in intracellular Ca^2+^ concentration by Ca^2+^ entry through Ca^2+^ release-activated Ca^2+^ (CRAC)/Orai1 induces the phosphatase calcineurin. As a result, the activated calcineurin dephosphorylates several serine residues of the NFAT. The NFAT then translocates to the nucleus where it binds to DNA and regulates target gene expression. Some ions or small molecules, including La^3+^, SKF96365, and 2-ABP, are able to inhibit the CRAC channel. Again, already-commercialized cyclosporine A and tacrolimus inhibit calcineurin. Since dysregulated Ca^2+^ signaling is involved in the pathogenesis of autoimmune diseases, intervention of Ca^2+^ signaling in store-operated Ca^2+^ entry (SOCE) through the Orai1–STIM1 pathway may be a promising approach to control autoimmune diseases.

In this review, we have discussed recent advances in understanding the role played by the Ca^2+^ signaling pathway in the function of diverse immune cells, especially lymphocytes. Normally, decreased Ca^2+^ concentration in ER stimulates STIM1, which can translocate to the membrane, and binds to Orai1. Consequently, SOCE is promoted, which triggers various cellular functions including cell proliferation, migration, and activation. As discussed above, the dysregulated Ca^2+^ signaling pathway, particularly in Th and B cells, is involved in the pathophysiology of autoimmune diseases, indicating that a therapeutic effect can be made in these diseases if the Ca^2+^-CRAC signaling pathway (from SOCE to the Orai1-STIM) is controlled (Figure 3). Moreover, inhibition of the Ca^2+^-calcineurin–NFAT pathway with cyclosporine A and tacrolimus has been the established treatment options of RA, lupus nephritis, and psoriasis patients who show an insufficient response to methotrexate. Therefore, CRAC channels in addition to the calcineurin–NFAT pathway are crucial to the lymphocyte function and development of autoimmunity, perhaps providing a new therapeutic target to treat human autoimmune diseases.

## Author Contributions

Y-JP, S-AY, MK, and W-UK conceptualized the article, reviewed the literature, and wrote the manuscript.

### Conflict of Interest

The authors declare that the research was conducted in the absence of any commercial or financial relationships that could be construed as a potential conflict of interest.

## References

[1] Feske S Wulff H Skolnik EY. Ion channels in innate and adaptive immunity. Annu Rev Immunol. (2015) 33:291-353. 10.1146/annurev-immunol-032414-112212 25861976 PMC4822408

[2] Gwack Y Feske S Srikanth S Hogan PG Rao A. Signalling to transcription: store-operated Ca2+ entry and NFAT activation in lymphocytes. Cell Calcium. (2007) 42:145–56. 10.1016/j.ceca.2007.03.007 17572487

[3] Maki BA Popescu GK. Extracellular Ca(2+) ions reduce NMDA receptor conductance and gating. J Gen Physiol. (2014) 144:379–92. 10.1085/jgp.201411244 25348411 PMC4210427

[4] Putney JW Jr. Formation and actions of calcium-mobilizing messenger, inositol 1,4,5-trisphosphate. Am J Physiol. (1987) 252:G149-57. 10.1152/ajpgi.1987.252.2.G149 3030126

[5] Negulescu PA Krasieva TB Khan A Kerschbaum HH Cahalan MD. Polarity of T cell shape, motility, and sensitivity to antigen. Immunity. (1996) 4:421–30. 10.1016/s1074-7613(00)80409-4 8630728

[6] Turner H Kinet JP. Signalling through the high-affinity IgE receptor Fc epsilonRI. Nature. (1999) 402:B24-30. 10.1038/35037021 10586892

[7] Pipkin ME Lieberman J. Delivering the kiss of death: progress on understanding how perforin works. Curr Opin Immunol. (2007) 19:301–8. 10.1016/j.coi.2007.04.011 17433871 PMC11484871

[8] Feske S. Calcium signalling in lymphocyte activation and disease. Nat Rev Immunol. (2007) 7:690–702. 10.1038/nri2152 17703229

[9] Ambudkar IS de Souza LB Ong HL. TRPC1, Orai1, and STIM1 in SOCE: Friends in tight spaces. Cell Calcium. (2017) 63:33–9. 10.1016/j.ceca.2016.12.009 28089266 PMC5466534

[10] Minke B. TRP channels and Ca2+ signaling. Cell Calcium. (2006) 40:261–75. 10.1016/j.ceca.2006.05.002 16806461 PMC1934411

[11] Wes PD Chevesich J Jeromin A Rosenberg C Stetten G Montell C. TRPC1, a human homolog of a Drosophila store-operated channel. Proc Natl Acad Sci USA. (1995) 92:9652–6. 10.1073/pnas.92.21.9652 7568191 PMC40860

[12] Chung MK Guler AD Caterina MJ. TRPV1 shows dynamic ionic selectivity during agonist stimulation. Nat Neurosci. (2008) 11:555–64. 10.1038/nn.2102 18391945

[13] Dong XP Wang X Xu H. TRP channels of intracellular membranes. J Neurochem. (2010) 113:313–28. 10.1111/j.1471-4159.2010.06626.x 20132470 PMC2905631

[14] Fenninger F Jefferies WA. What's bred in the bone: calcium channels in lymphocytes. J Immunol. (2019) 202:1021–30. 10.4049/jimmunol.1800837 30718290

[15] Cheng KT Liu X Ong HL Swaim W Ambudkar IS. Local Ca(2)+ entry via Orai1 regulates plasma membrane recruitment of TRPC1 and controls cytosolic Ca(2)+ signals required for specific cell functions. PLoS Biol. (2011) 9:e1001025. 10.1371/journal.pbio.1001025 21408196 PMC3050638

[16] Huang GN Zeng W Kim JY Yuan JP Han L Muallem S. STIM1 carboxyl-terminus activates native SOC, I(crac) and TRPC1 channels. Nat Cell Biol. (2006) 8:1003–10. 10.1038/ncb1454 16906149

[17] Yuan JP Zeng W Huang GN Worley PF Muallem S. STIM1 heteromultimerizes TRPC channels to determine their function as store-operated channels. Nat Cell Biol. (2007) 9:636–45. 10.1038/ncb1590 17486119 PMC2699187

[18] Liou J Kim ML Heo WD Jones JT Myers JW Ferrell JE Jr. STIM is a Ca2+ sensor essential for Ca2+-store-depletion-triggered Ca2+ influx. Curr Biol. (2005) 15:1235–41. 10.1016/j.cub.2005.05.055 16005298 PMC3186072

[19] Putney JW Jr. A model for receptor-regulated calcium entry. Cell Calcium. (1986) 7:1–12. 10.1016/0143-4160(86)90026-6 2420465

[20] Randriamampita C Tsien RY. Emptying of intracellular Ca2+ stores releases a novel small messenger that stimulates Ca2+ influx. Nature. (1993) 364:809–14. 10.1038/364809a0 8355806

[21] Patterson RL van Rossum DB Gill DL. Store-operated Ca2+ entry: evidence for a secretion-like coupling model. Cell. (1999) 98:487–99. 10.1016/s0092-8674(00)81977-7 10481913

[22] Roos J DiGregorio PJ Yeromin AV Ohlsen K Lioudyno M Zhang S. STIM1, an essential and conserved component of store-operated Ca2+ channel function. J Cell Biol. (2005) 169:435–45. 10.1083/jcb.200502019 15866891 PMC2171946

[23] Williams RT Manji SS Parker NJ Hancock MS Van Stekelenburg L Eid JP. Identification and characterization of the STIM (stromal interaction molecule) gene family: coding for a novel class of transmembrane proteins. Biochem J. (2001) 357:673–85. 10.1042/0264-6021:3570673 11463338 PMC1221997

[24] Zhang SL Yu Y Roos J Kozak JA Deerinck TJ Ellisman MH. STIM1 is a Ca2+ sensor that activates CRAC channels and migrates from the Ca2+ store to the plasma membrane. Nature. (2005) 437:902–5. 10.1038/nature04147 16208375 PMC1618826

[25] Oh-Hora M Yamashita M Hogan PG Sharma S Lamperti E Chung W. Dual functions for the endoplasmic reticulum calcium sensors STIM1 and STIM2 in T cell activation and tolerance. Nat Immunol. (2008) 9:432–43. 10.1038/ni1574 18327260 PMC2737533

[26] Liou J Fivaz M Inoue T Meyer T. Live-cell imaging reveals sequential oligomerization and local plasma membrane targeting of stromal interaction molecule 1 after Ca2+ store depletion. Proc Natl Acad Sci USA. (2007) 104:9301–6. 10.1073/pnas.0702866104 17517596 PMC1890489

[27] Manji SS Parker NJ Williams RT van Stekelenburg L Pearson RB Dziadek M. STIM1: a novel phosphoprotein located at the cell surface. Biochim Biophys Acta. (2000) 1481:147–55. 10.1016/s0167-4838(00)00105-9 11004585

[28] Baba Y Hayashi K Fujii Y Mizushima A Watarai H Wakamori M. Coupling of STIM1 to store-operated Ca2+ entry through its constitutive and inducible movement in the endoplasmic reticulum. Proc Natl Acad Sci USA. (2006) 103:16704–9. 10.1073/pnas.0608358103 17075073 PMC1636519

[29] Luik RM Wu MM Buchanan J Lewis RS. The elementary unit of store-operated Ca2+ entry: local activation of CRAC channels by STIM1 at ER-plasma membrane junctions. J Cell Biol. (2006) 174:815–25. 10.1083/jcb.200604015 16966423 PMC2064336

[30] Brandman O Liou J Park WS Meyer T. STIM2 is a feedback regulator that stabilizes basal cytosolic and endoplasmic reticulum Ca2+ levels. Cell. (2007) 131:1327–39. 10.1016/j.cell.2007.11.039 18160041 PMC2680164

[31] Parvez S Beck A Peinelt C Soboloff J Lis A Monteilh-Zoller M. STIM2 protein mediates distinct store-dependent and store-independent modes of CRAC channel activation. FASEB J. (2008) 22:752–61. 10.1096/fj.07-9449com 17905723 PMC3601890

[32] Berna-Erro A Braun A Kraft R Kleinschnitz C Schuhmann MK Stegner D. STIM2 regulates capacitive Ca2+ entry in neurons and plays a key role in hypoxic neuronal cell death. Sci Signal. (2009) 2:ra67. 10.1126/scisignal.2000522 19843959

[33] Feske S Gwack Y Prakriya M Srikanth S Puppel SH Tanasa B. A mutation in Orai1 causes immune deficiency by abrogating CRAC channel function. Nature. (2006) 441:179–85. 10.1038/nature04702 16582901

[34] Vig M Peinelt C Beck A Koomoa DL Rabah D Koblan-Huberson M. CRACM1 is a plasma membrane protein essential for store-operated Ca2+ entry. Science. (2006) 312:1220–3. 10.1126/science.1127883 16645049 PMC5685805

[35] Prakriya M Feske S Gwack Y Srikanth S Rao A Hogan PG. Orai1 is an essential pore subunit of the CRAC channel. Nature. (2006) 443:230–3. 10.1038/nature05122 16921383

[36] Amcheslavsky A Wood ML Yeromin AV Parker I Freites JA Tobias DJ. Molecular biophysics of Orai store-operated Ca2+ channels. Biophys J. (2015) 108:237–46. 10.1016/j.bpj.2014.11.3473 25606672 PMC4302196

[37] Yamashita M Navarro-Borelly L McNally BA Prakriya M. Orai1 mutations alter ion permeation and Ca2+-dependent fast inactivation of CRAC channels: evidence for coupling of permeation and gating. J Gen Physiol. (2007) 130:525–40. 10.1085/jgp.200709872 17968026 PMC2151669

[38] Yeromin AV Zhang SL Jiang W Yu Y Safrina O Cahalan MD. Molecular identification of the CRAC channel by altered ion selectivity in a mutant of Orai. Nature. (2006) 443:226–9. 10.1038/nature05108 16921385 PMC2756048

[39] Soboloff J Spassova MA Tang XD Hewavitharana T Xu W Gill DL. Orai1 and STIM reconstitute store-operated calcium channel function. J Biol Chem. (2006) 281:20661–5. 10.1074/jbc.C600126200 16766533

[40] Hogan PG Chen L Nardone J Rao A. Transcriptional regulation by calcium, calcineurin, and NFAT. Genes Dev. (2003) 17:2205–32. 10.1101/gad.1102703 12975316

[41] Macian F. NFAT proteins: key regulators of T-cell development and function. Nat Rev Immunol. (2005) 5:472–84. 10.1038/nri1632 15928679

[42] Horsley V Pavlath GK. NFAT: ubiquitous regulator of cell differentiation and adaptation. J Cell Biol. (2002) 156:771–4. 10.1083/jcb.200111073 11877454 PMC2173310

[43] Park S Uesugi M Verdine GL. A second calcineurin binding site on the NFAT regulatory domain. Proc Natl Acad Sci USA. (2000) 97:7130–5. 10.1073/pnas.97.13.7130 10860980 PMC16511

[44] Oukka M Ho IC de la Brousse FC Hoey T Grusby MJ Glimcher LH. The transcription factor NFAT4 is involved in the generation and survival of T cells. Immunity. (1998) 9:295–304. 10.1016/s1074-7613(00)80612-3 9768749

[45] Amasaki Y Masuda ES Imamura R Arai K Arai N. Distinct NFAT family proteins are involved in the nuclear NFAT-DNA binding complexes from human thymocyte subsets. J Immunol. (1998) 160:2324–33. 9498773

[46] Kar P Mirams GR Christian HC Parekh AB Control of NFAT Isoform Activation and NFAT-Dependent Gene Expression through Two Coincident and Spatially Segregated Intracellular Ca(2+) Signals. Mol Cell. (2016) 64:746–59. 10.1016/j.molcel.2016.11.011 27863227 PMC5128683

[47] Davies EV Hallett MB. Cytosolic Ca2+ signalling in inflammatory neutrophils: implications for rheumatoid arthritis (Review). Int J Mol Med. (1998) 1:485–90. 10.3892/ijmm.1.2.485 9852254

[48] Jaconi ME Theler JM Schlegel W Appel RD Wright SD Lew PD. Multiple elevations of cytosolic-free Ca2+ in human neutrophils: initiation by adherence receptors of the integrin family. J Cell Biol. (1991) 112:1249–57. 10.1083/jcb.112.6.1249 1900302 PMC2288892

[49] Pettit EJ Hallett MB. Release of 'caged' cytosolic Ca2+ triggers rapid spreading of human neutrophils adherent via integrin engagement. J Cell Sci. (1998) 111 (Pt 15):2209-15. 9664042 10.1242/jcs.111.15.2209

[50] Bengtsson T Jaconi ME Gustafson M Magnusson KE Theler JM Lew DP. Actin dynamics in human neutrophils during adhesion and phagocytosis is controlled by changes in intracellular free calcium. Eur J Cell Biol. (1993) 62:49–58. 8269978

[51] Downey GP Chan CK Trudel S Grinstein S. Actin assembly in electropermeabilized neutrophils: role of intracellular calcium. J Cell Biol. (1990) 110:1975–82. 10.1083/jcb.110.6.1975 2112547 PMC2116117

[52] Lew PD Monod A Waldvogel FA Dewald B Baggiolini M Pozzan T. Quantitative analysis of the cytosolic free calcium dependency of exocytosis from three subcellular compartments in intact human neutrophils. J Cell Biol. (1986) 102:2197–204. 10.1083/jcb.102.6.2197 3011810 PMC2114244

[53] Brechard S Plancon S Melchior C Tschirhart EJ STIM1 but not STIM2 is an essential regulator of Ca2+ influx-mediated NADPH oxidase activity in neutrophil-like HL-60 cells. Biochem Pharmacol. (2009) 78:504–13. 10.1016/j.bcp.2009.05.006 19433064

[54] Zou W Meng X Cai C Zou M Tang S Chu X. Store-operated Ca2+ entry (SOCE) plays a role in the polarization of neutrophil-like HL-60 cells by regulating the activation of Akt, Src, and Rho family GTPases. Cell Physiol Biochem. (2012) 30:221–37. 10.1159/000339059 22759969

[55] Nunes P Cornut D Bochet V Hasler U Oh-Hora M Waldburger JM. STIM1 juxtaposes ER to phagosomes, generating Ca(2)(+) hotspots that boost phagocytosis. Curr Biol. (2012) 22:1990–7. 10.1016/j.cub.2012.08.049 23041196

[56] Steinckwich N Schenten V Melchior C Brechard S Tschirhart EJ. An essential role of STIM1, Orai1, and S100A8-A9 proteins for Ca2+ signaling and FcgammaR-mediated phagosomal oxidative activity. J Immunol. (2011) 186:2182–91. 10.4049/jimmunol.1001338 21239714

[57] Brechard S Melchior C Plancon S Schenten V Tschirhart EJ. Store-operated Ca2+ channels formed by TRPC1, TRPC6 and Orai1 and non-store-operated channels formed by TRPC3 are involved in the regulation of NADPH oxidase in HL-60 granulocytes. Cell Calcium. (2008) 44:492–506. 10.1016/j.ceca.2008.03.002 18436303

[58] Steinckwich N Myers P Janardhan KS Flagler ND King D Petranka JG. Role of the store-operated calcium entry protein, STIM1, in neutrophil chemotaxis and infiltration into a murine model of psoriasis-inflamed skin. FASEB J. (2015) 29:3003–13. 10.1096/fj.14-265215 25837581 PMC4478797

[59] Zhang H Clemens RA Liu F Hu Y Baba Y Theodore P. STIM1 calcium sensor is required for activation of the phagocyte oxidase during inflammation and host defense. Blood. (2014) 123:2238–49. 10.1182/blood-2012-08-450403 24493668 PMC3975260

[60] Elling R Keller B Weidinger C Haffner M Deshmukh SD Zee I. Preserved effector functions of human ORAI1- and STIM1-deficient neutrophils. J Allergy Clin Immunol. (2016) 137:1587–91.e7. 10.1016/j.jaci.2015.09.047 26670474 PMC4860117

[61] Teti A Blair HC Schlesinger P Grano M Zambonin-Zallone A Kahn AJ. Extracellular protons acidify osteoclasts, reduce cytosolic calcium, and promote expression of cell-matrix attachment structures. J Clin Invest. (1989) 84:773–80. 10.1172/jci114235 2547838 PMC329718

[62] Mentaverri R Yano S Chattopadhyay N Petit L Kifor O Kamel S. The calcium sensing receptor is directly involved in both osteoclast differentiation and apoptosis. FASEB J. (2006) 20:2562–4. 10.1096/fj.06-6304fje 17077282

[63] Yang S Li YP. RGS10-null mutation impairs osteoclast differentiation resulting from the loss of [Ca2+]i oscillation regulation. Genes Dev. (2007) 21:1803–16. 10.1101/gad.1544107 17626792 PMC1920174

[64] Li P Bian X Liu C Wang S Guo M Tao Y. STIM1 and TRPV4 regulate fluid flow-induced calcium oscillation at early and late stages of osteoclast differentiation. Cell Calcium. (2018) 71:45–52. 10.1016/j.ceca.2017.12.001 29604963

[65] Kajiya H Okamoto F Nemoto T Kimachi K Toh-Goto K Nakayana S. RANKL-induced TRPV2 expression regulates osteoclastogenesis via calcium oscillations. Cell Calcium. (2010) 48:260–9. 10.1016/j.ceca.2010.09.010 20980052

[66] Hwang SY Putney JW. Orai1-mediated calcium entry plays a critical role in osteoclast differentiation and function by regulating activation of the transcription factor NFATc1. FASEB J. (2012) 26:1484–92. 10.1096/fj.11-194399 22198385 PMC3316896

[67] van der Eerden BC Hoenderop JG de Vries TJ Schoenmaker T Buurman CJ Uitterlinden AG. The epithelial Ca2+ channel TRPV5 is essential for proper osteoclastic bone resorption. Proc Natl Acad Sci USA. (2005) 102:17507–12. 10.1073/pnas.0505789102 16291808 PMC1297662

[68] Takayanagi H Kim S Koga T Nishina H Isshiki M Yoshida H. Induction and activation of the transcription factor NFATc1 (NFAT2) integrate RANKL signaling in terminal differentiation of osteoclasts. Dev Cell. (2002) 3:889–901. 10.1016/s1534-5807(02)00369-6 12479813

[69] Chamoux E Bisson M Payet MD Roux S. TRPV-5 mediates a receptor activator of NF-kappaB (RANK) ligand-induced increase in cytosolic Ca2+ in human osteoclasts and down-regulates bone resorption. J Biol Chem. (2010) 285:25354–62. 10.1074/jbc.M109.075234 20547482 PMC2919098

[70] Masuyama R Vriens J Voets T Karashima Y Owsianik G Vennekens R. TRPV4-mediated calcium influx regulates terminal differentiation of osteoclasts. Cell Metab. (2008) 8:257–65. 10.1016/j.cmet.2008.08.002 18762026

[71] Bhakta NR Oh DY Lewis RS. Calcium oscillations regulate thymocyte motility during positive selection in the three-dimensional thymic environment. Nat Immunol. (2005) 6:143–51. 10.1038/ni1161 15654342

[72] Oh-hora M. Calcium signaling in the development and function of T-lineage cells. Immunol Rev. (2009) 231:210–24. 10.1111/j.1600-065X.2009.00819.x 19754899

[73] Nakayama T Ueda Y Yamada H Shores EW Singer A June CH. In vivo calcium elevations in thymocytes with T cell receptors that are specific for self ligands. Science. (1992) 257:96–9. 10.1126/science.1621102 1621102

[74] Picard C McCarl CA Papolos A Khalil S Luthy K Hivroz C. STIM1 mutation associated with a syndrome of immunodeficiency and autoimmunity. N Engl J Med. (2009) 360:1971–80. 10.1056/NEJMoa0900082 19420366 PMC2851618

[75] Weber KS Miller MJ Allen PM. Th17 cells exhibit a distinct calcium profile from Th1 and Th2 cells and have Th1-like motility and NF-AT nuclear localization. J Immunol. (2008) 180:1442–50. 10.4049/jimmunol.180.3.1442 18209039

[76] Klareskog L Stolt P Lundberg K Kallberg H Bengtsson C Grunewald J. A new model for an etiology of rheumatoid arthritis: smoking may trigger HLA-DR (shared epitope)-restricted immune reactions to autoantigens modified by citrullination. Arthritis Rheum. (2006) 54:38–46. 10.1002/art.21575 16385494

[77] Edwards JC Cambridge G. B-cell targeting in rheumatoid arthritis and other autoimmune diseases. Nat Rev Immunol. (2006) 6:394–403. 10.1038/nri1838 16622478

[78] Firestein GS. Evolving concepts of rheumatoid arthritis. Nature. (2003) 423:356–61. 10.1038/nature01661 12748655

[79] Weyand CM Goronzy JJ. T-cell-targeted therapies in rheumatoid arthritis. Nat Clin Pract Rheumatol. (2006) 2:201–10. 10.1038/ncprheum0142 16932686

[80] Masuda K Masuda R Neidhart M Simmen BR Michel BA Muller-Ladner U. Molecular profile of synovial fibroblasts in rheumatoid arthritis depends on the stage of proliferation. Arthritis Res. (2002) 4:R8. 10.1186/ar427 12223111 PMC125298

[81] Sakaguchi N Takahashi T Hata H Nomura T Tagami T Yamazaki S. Altered thymic T-cell selection due to a mutation of the ZAP-70 gene causes autoimmune arthritis in mice. Nature. (2003) 426:454–60. 10.1038/nature02119 14647385

[82] Yen JH Chang CM Hsu YW Lee CH Wu MS Hwang DY. A polymorphism of ORAI1 rs7135617, is associated with susceptibility to rheumatoid arthritis. Mediators Inflamm. (2014) 2014:834831. 10.1155/2014/834831 24808640 PMC3997980

[83] Liu S Watanabe S Shudou M Kuno M Miura H Maeyama K. Upregulation of store-operated Ca(2+) entry in the naive CD4(+) T cells with aberrant cytokine releasing in active rheumatoid arthritis. Immunol Cell Biol. (2014) 92:752–60. 10.1038/icb.2014.45 24935456

[84] Miskin JE Abrams CC Goatley LC Dixon LK. A viral mechanism for inhibition of the cellular phosphatase calcineurin. Science. (1998) 281:562–5. 10.1126/science.281.5376.562 9677199

[85] Yoo SA Park BH Park GS Koh HS Lee MS Ryu SH. Calcineurin is expressed and plays a critical role in inflammatory arthritis. J Immunol. (2006) 177:2681–90. 10.4049/jimmunol.177.4.2681 16888030

[86] Yoo SA Park BH Yoon HJ Lee JY Song JH Kim HA. Calcineurin modulates the catabolic and anabolic activity of chondrocytes and participates in the progression of experimental osteoarthritis. Arthritis Rheum. (2007) 56:2299–311. 10.1002/art.22731 17599750

[87] Wells G Haguenauer D Shea B Suarez-Almazor ME Welch VA Tugwell P Cyclosporine for rheumatoid arthritis. Cochrane Database Syst Rev. (2000):Cd001083. 10.1002/14651858.Cd001083 PMC8406939 10796412

[88] Zaichuk TA Shroff EH Emmanuel R Filleur S Nelius T Volpert OV. Nuclear factor of activated T cells balances angiogenesis activation and inhibition. J Exp Med. (2004) 199:1513–22. 10.1084/jem.20040474 15184502 PMC2211785

[89] Hernandez GL Volpert OV Iniguez MA Lorenzo E Martinez-Martinez S Grau R. Selective inhibition of vascular endothelial growth factor-mediated angiogenesis by cyclosporin A: roles of the nuclear factor of activated T cells and cyclooxygenase 2. J Exp Med. (2001) 193:607–20. 10.1084/jem.193.5.607 11238591 PMC2193389

[90] Liossis SN Kovacs B Dennis G Kammer GM Tsokos GC. B cells from patients with systemic lupus erythematosus display abnormal antigen receptor-mediated early signal transduction events. J Clin Invest. (1996) 98:2549–57. 10.1172/jci119073 8958217 PMC507712

[91] Enyedy EJ Mitchell JP Nambiar MP Tsokos GC. Defective FcgammaRIIb1 signaling contributes to enhanced calcium response in B cells from patients with systemic lupus erythematosus. Clin Immunol. (2001) 101:130–5. 10.1006/clim.2001.5104 11683571

[92] Hippen KL Buhl AM D'Ambrosio D Nakamura K Persin C Cambier JC. Fc gammaRIIB1 inhibition of BCR-mediated phosphoinositide hydrolysis and Ca2+ mobilization is integrated by CD19 dephosphorylation. Immunity. (1997) 7:49–58. 10.1016/s1074-7613(00)80509-9 9252119

[93] Hibbs ML Tarlinton DM Armes J Grail D Hodgson G Maglitto R. Multiple defects in the immune system of Lyn-deficient mice, culminating in autoimmune disease. Cell. (1995) 83:301–11. 10.1016/0092-8674(95)90171-x 7585947

[94] Yu P Constien R Dear N Katan M Hanke P Bunney TD. Autoimmunity and inflammation due to a gain-of-function mutation in phospholipase C gamma 2 that specifically increases external Ca2+ entry. Immunity. (2005) 22:451–65. 10.1016/j.immuni.2005.01.018 15845450

[95] Sato S Miller AS Inaoki M Bock CB Jansen PJ Tang ML. CD22 is both a positive and negative regulator of B lymphocyte antigen receptor signal transduction: altered signaling in CD22-deficient mice. Immunity. (1996) 5:551–62. 10.1016/s1074-7613(00)80270-8 8986715

[96] Rother N van der Vlag J. Disturbed T Cell Signaling and Altered Th17 and Regulatory T Cell Subsets in the Pathogenesis of Systemic Lupus Erythematosus. Front Immunol. (2015) 6:610. 10.3389/fimmu.2015.00610 26648939 PMC4663269

[97] Akahoshi M Nakashima H Tanaka Y Kohsaka T Nagano S Ohgami E. Th1/Th2 balance of peripheral T helper cells in systemic lupus erythematosus. Arthritis Rheum. (1999) 42:1644–8. 10.1002/1529-0131(199908)42:8<1644::AID-ANR12>3.0.CO;2-L 10446863

[98] Kyttaris VC Wang Y Juang YT Weinstein A Tsokos GC. Increased levels of NF-ATc2 differentially regulate CD154 and IL-2 genes in T cells from patients with systemic lupus erythematosus. J Immunol. (2007) 178:1960–6. 10.4049/jimmunol.178.3.1960 17237447

[99] Desai-Mehta A Lu L Ramsey-Goldman R Datta SK. Hyperexpression of CD40 ligand by B and T cells in human lupus and its role in pathogenic autoantibody production. J Clin Invest. (1996) 97:2063–73. 10.1172/jci118643 8621796 PMC507281

[100] Iezzi G Sonderegger I Ampenberger F Schmitz N Marsland BJ Kopf M. CD40-CD40L cross-talk integrates strong antigenic signals and microbial stimuli to induce development of IL-17-producing CD4+ T cells. Proc Natl Acad Sci USA. (2009) 106:876–81. 10.1073/pnas.0810769106 19136631 PMC2630101

[101] Crispin JC Oukka M Bayliss G Cohen RA Van Beek CA Stillman IE. Expanded double negative T cells in patients with systemic lupus erythematosus produce IL-17 and infiltrate the kidneys. J Immunol. (2008) 181:8761–6. 10.4049/jimmunol.181.12.8761 19050297 PMC2596652

[102] Kyttaris VC Zhang Z Kampagianni O Tsokos GC. Calcium signaling in systemic lupus erythematosus T cells: a treatment target. Arthritis Rheum. (2011) 63:2058–66. 10.1002/art.30353 21437870 PMC3128171

[103] Faul C Donnelly M Merscher-Gomez S Chang YH Franz S Delfgaauw J. The actin cytoskeleton of kidney podocytes is a direct target of the antiproteinuric effect of cyclosporine A. Nat Med. (2008) 14:931–8. 10.1038/nm.1857 18724379 PMC4109287

[104] Liao R Liu Q Zheng Z Fan J Peng W Kong Q. Tacrolimus protects podocytes from injury in lupus nephritis partly by stabilizing the cytoskeleton and inhibiting podocyte apoptosis. PLoS ONE. (2015) 10:e0132724. 10.1371/journal.pone.0132724 26161538 PMC4498640

[105] Larsson A Bredberg A Henriksson G Manthorpe R Sallmyr A. Immunohistochemistry of the B-cell component in lower lip salivary glands of Sjogren's syndrome and healthy subjects. Scand J Immunol. (2005) 61:98–107. 10.1111/j.0300-9475.2005.01540.x 15644129

[106] Cheng KT Alevizos I Liu X Swaim WD Yin H Feske S. STIM1 and STIM2 protein deficiency in T lymphocytes underlies development of the exocrine gland autoimmune disease, Sjogren's syndrome. Proc Natl Acad Sci USA. (2012) 109:14544–9. 10.1073/pnas.1207354109 22904194 PMC3437853

[107] Kedor C Zernicke J Hagemann A Gamboa LM Callhoff J Burmester GR. A phase II investigator-initiated pilot study with low-dose cyclosporine A for the treatment of articular involvement in primary Sjogren's syndrome. Clin Rheumatol. (2016) 35:2203–10. 10.1007/s10067-016-3360-4 27470087

[108] Schon MP Boehncke WH. Psoriasis. N Engl J Med. (2005) 352:1899–912. 10.1056/NEJMra041320 15872205

[109] Quatresooz P Hermanns-Le T Pierard GE Humbert P Delvenne P Pierard-Franchimont C. Ustekinumab in psoriasis immunopathology with emphasis on the Th17-IL23 axis: a primer. J Biomed Biotechnol. (2012) 2012:147413. 10.1155/2012/147413 22754278 PMC3384985

[110] Nestle FO Kaplan DH Barker J. Psoriasis. N Engl J Med. (2009) 361:496–509. 10.1056/NEJMra0804595 19641206

[111] Karczewski J Dobrowolska A Rychlewska-Hanczewska A Adamski Z. New insights into the role of T cells in pathogenesis of psoriasis and psoriatic arthritis. Autoimmunity. (2016) 49:435–50. 10.3109/08916934.2016.1166214 27050731

[112] Schlaak JF Buslau M Jochum W Hermann E Girndt M Gallati H. T cells involved in psoriasis vulgaris belong to the Th1 subset. J Invest Dermatol. (1994) 102:145–9. 10.1111/1523-1747.ep12371752 8106745

[113] Richetta AG Mattozzi C Salvi M Giancristoforo S D'Epiro S Milana B. CD4+ CD25+ T-regulatory cells in psoriasis. Correlation between their numbers and biologics-induced clinical improvement. Eur J Dermatol. (2011) 21:344–8. 10.1684/ejd.2011.1362 21680285

[114] Serrano CJ Graham L DeBell K Rawat R Veri MC Bonvini E. A new tyrosine phosphorylation site in PLC gamma 1: the role of tyrosine 775 in immune receptor signaling. J Immunol. (2005) 174:6233–7. 10.4049/jimmunol.174.10.6233 15879121

[115] Schlapbach C Gehad A Yang C Watanabe R Guenova E Teague JE. Human TH9 cells are skin-tropic and have autocrine and paracrine proinflammatory capacity. Sci Transl Med. (2014) 6:219ra8. 10.1126/scitranslmed.3007828 24431112 PMC4102325

[116] Nowak EC Weaver CT Turner H Begum-Haque S Becher B Schreiner B. IL-9 as a mediator of Th17-driven inflammatory disease. J Exp Med. (2009) 206:1653–60. 10.1084/jem.20090246 19596803 PMC2722185

[117] Tsuda K Yamanaka K Kitagawa H Akeda T Naka M Niwa K. Calcineurin inhibitors suppress cytokine production from memory T cells and differentiation of naive T cells into cytokine-producing mature T cells. PLoS ONE. (2012) 7:e31465. 10.1371/journal.pone.0031465 22359594 PMC3281079

[118] Lew W Bowcock AM Krueger JG. Psoriasis vulgaris: cutaneous lymphoid tissue supports T-cell activation and “Type 1” inflammatory gene expression. Trends Immunol. (2004) 25:295–305. 10.1016/j.it.2004.03.006 15145319

[119] Haider AS Lowes MA Suarez-Farinas M Zaba LC Cardinale I Khatcherian A Identification of cellular pathways of “type 1,” Th17 T cells, and TNF- and inducible nitric oxide synthase-producing dendritic cells in autoimmune inflammation through pharmacogenomic study of cyclosporine A in psoriasis. J Immunol. (2008) 180:1913–20. 10.4049/jimmunol.180.3.1913 18209089

[120] Northrop JP Ho SN Chen L Thomas DJ Timmerman LA Nolan GP. NF-AT components define a family of transcription factors targeted in T-cell activation. Nature. (1994) 369:497–502. 10.1038/369497a0 8202141

[121] Nishio H Matsui K Tsuji H Tamura A Suzuki K. Immunolocalization of calcineurin and FKBP12, the FK506-binding protein, in Hassall'scorpuscles of human thymus and epidermis. Histochem Cell Biol. (2000) 114:9–14. 10.1007/s004180000168 10959817

[122] Furue M Katz SI. The effect of cyclosporine on epidermal cells. I. Cyclosporine inhibits accessory cell functions of epidermal Langerhans cells *in vitro*. J Immunol. (1988) 140:4139–43. 3259608

[123] Prens EP van Joost T Hegmans JP t Hooft-Benne K Ysselmuiden OE Benner R. Effects of cyclosporine on cytokines and cytokine receptors in psoriasis. J Am Acad Dermatol. (1995) 33:947–53. 10.1016/0190-9622(95)90285-6 7490364

[124] Soleymani T Vassantachart JM Wu JJ. Comparison of guidelines for the use of cyclosporine for psoriasis: a critical appraisal and comprehensive review. J Drugs Dermatol. (2016) 15:293–301. 26954314

[125] Numaga-Tomita T Putney JW. Role of STIM1- and Orai1-mediated Ca2+ entry in Ca2+-induced epidermal keratinocyte differentiation. J Cell Sci. (2013) 126:605–12. 10.1242/jcs.115980 23203806 PMC3613182

[126] Menon GK Elias PM. Ultrastructural localization of calcium in psoriatic and normal human epidermis. Arch Dermatol. (1991) 127:57–63. 1986708

[127] Karvonen SL Korkiamaki T Yla-Outinen H Nissinen M Teerikangas H Pummi K. Psoriasis and altered calcium metabolism: downregulated capacitative calcium influx and defective calcium-mediated cell signaling in cultured psoriatic keratinocytes. J Invest Dermatol. (2000) 114:693–700. 10.1046/j.1523-1747.2000.00926.x 10733675

[128] Leuner K Kraus M Woelfle U Beschmann H Harteneck C Boehncke WH. Reduced TRPC channel expression in psoriatic keratinocytes is associated with impaired differentiation and enhanced proliferation. PLoS ONE. (2011) 6:e14716. 10.1371/journal.pone.0014716 21364982 PMC3043053

[129] Leuner K Kazanski V Muller M Essin K Henke B Gollasch M. Hyperforin–a key constituent of St. John's wort specifically activates TRPC6 channels. FASEB J. (2007) 21:4101–11. 10.1096/fj.07-8110com 17666455

[130] Tian C Du L Zhou Y Li M. Store-operated CRAC channel inhibitors: opportunities and challenges. Future Med Chem. (2016) 8:817–32. 10.4155/fmc-2016-0024 27149324 PMC5558521

[131] Robinson AP Harp CT Noronha A Miller SD. The experimental autoimmune encephalomyelitis (EAE) model of MS: utility for understanding disease pathophysiology and treatment. Handb Clin Neurol. (2014) 122:173–89. 10.1016/b978-0-444-52001-2.00008-x 24507518 PMC3981554

[132] Schuhmann MK Stegner D Berna-Erro A Bittner S Braun A Kleinschnitz C. Stromal interaction molecules 1 and 2 are key regulators of autoreactive T cell activation in murine autoimmune central nervous system inflammation. J Immunol. (2010) 184:1536–42. 10.4049/jimmunol.0902161 20028655

[133] Kaufmann U Shaw PJ Kozhaya L Subramanian R Gaida K Unutmaz D Selective ORAI1 inhibition ameliorates autoimmune central nervous system inflammation by suppressing effector but not regulatory T cell function. J Immunol. (2016) 196:573–85. 10.4049/jimmunol.1501406 26673135 PMC4707123

[134] Matsumoto M Fujii Y Baba A Hikida M Kurosaki T Baba Y. The calcium sensors STIM1 and STIM2 control B cell regulatory function through interleukin-10 production. Immunity. (2011) 34:703–14. 10.1016/j.immuni.2011.03.016 21530328

